# Multimethod Assessment of the Cyclic Fatigue Strength of ProGlider, Edge Glide Path and R-Pilot Endodontic Instruments

**DOI:** 10.3390/dj10020030

**Published:** 2022-02-17

**Authors:** Jorge N. R. Martins, Duarte Marques, Isabel Vasconcelos, Sofia Arantes-Oliveira, João Caramês, Francisco Manuel Braz Fernandes

**Affiliations:** 1 Faculdade de Medicina Dentária, Universidade de Lisboa, 1600-277 Lisboa, Portugal; duarte.marques@campus.ul.pt (D.M.); isabelvasconcelos@campus.ul.pt (I.V.); sofiaaol@campus.ul.pt (S.A.-O.); joaocarames@institutodeimplantologia.pt (J.C.); 2 Centro de Estudo de Medicina Dentária Baseada na Evidência (CEMDBE), 1600-277 Lisboa, Portugal; 3 Implantology Institute, 1070-064 Liaboa, Portugal; 4 LIBPhys-FCT UID/FIS/04559/2013, 1600-277 Lisboa, Portugal; 5 Center for Rapid and Sustainable Product Development, Polytechnic Institute of Leiria, 2411-901 Leiria, Portugal; 6 CENIMAT/I3N, Department of Materials Science, NOVA School of Science and Technology, Universidade NOVA de Lisboa, 2829-516 Caparica, Portugal; fbf@fct.unl.pt

**Keywords:** cyclic fatigue, differential scanning calorimetry, endodontics, energy-dispersive X-ray spectroscopy, scanning electron microscopy

## Abstract

Background: The aim of this study was to comprehensively evaluate the cyclic fatigue strength of ProGlider, Edge Glide Path, and R-Pilot instruments. Methods: Sixty-three instruments were submitted to a multimethod evaluation. Their design was analyzed by stereomicroscopy and scanning electron microscopy, including the number of blades, helical angle means, cross-sectional design, surface finishing, and symmetry. Energy-dispersive X-ray spectroscopy was used determine the nickel/titanium elements ratio, and differential scanning calorimetry determined the instruments’ phase transformation temperatures. The cyclic fatigue tests were conducted in an artificial canal with a 6 mm radius and 86 degrees of curvature. The Mood’s median test and one-way ANOVA were used to determine differences, with the significance level set at 0.05. Results: The ProGlider presented the highest number of blades (*n* = 21), while R-Pilot had the highest helical angles (26.4°). Differences were noted in the instruments’ cross-sections and surface finishing. The ProGlider and R-Pilot showed some similarities regarding the phase transformation temperatures but differed from the Edge Glide Path. All alloys showed an almost equiatomic nickel/titanium ratio. The R-Pilot instruments showed a significantly higher (*p* < 0.05) time to fracture than both the other files. Conclusion: Reciprocating R-Pilot instruments showed a higher cyclic fatigue time to fracture than the ProGlider and Edge Glide Path rotary files.

## 1. Introduction

Root canal preparation procedures are among the most technically challenging steps in root canal therapy. The introduction of nickel–titanium (NiTi) instruments has improved the feasibility of root canal treatment in clinical practice but they cannot remove all the difficulties or procedural complications potentially created during the mechanical preparation of the root canal system [1]. Although uncommon, iatrogenic errors and complications, such as ledge formation, root perforation, apical zipping, or instrument separation may occur and can influence the treatment prognosis [1,2].

Two modes of separation failure have been identified in NiTi instruments: torsional and cyclic fatigue failures [3]. The torsional failure mode is usually associated with files that have been submitted to intense twisting stress for a short time, corresponding to a high-stress condition capable of exceeding the 8% strain withstood by the NiTi alloy [4]. The instrument getting jammed inside a root canal of small diameter while the shank continues to rotate might be the most common cause of this type of failure [5,6]. The cyclic fatigue failure mode tends to occur when an instrument is submitted to repetitive cycles of tension and compression, as happens in the preparation of severely curved root canals, or due to overuse [3]. Scanning electron microscopy can reveal microcracks in the alloy structure, which may reflect an irreversible process of crack propagation [7,8].

Multiple studies have been conducted over the years to assess the factors that can potentiate both types of failure, and which conditions could minimize them. Regarding the cyclic fatigue strength in particular, conducting the instrumentation with in-and-out continuous motions [9], oscillatory asymmetric kinematics [10,11], and using thermally-treated NiTi instruments [12] are among the conditions that tend to increase the endodontic files’ cyclic fatigue time to fracture. Additionally, instruments previously submitted to flexural stress tend to present a decrease in both the maximum torque before fracture and the angle of rotation when compared to new ones [13]. Another relevant factor that can reduce the risk of instrument separation is creating an initial glide path prior to mechanical enlargement of the root canal [14]. Initially, the instruments advocated for this clinical step were small stainless-steel hand files, but lately, mechanized NiTi glide path instruments have been suggested. Among the most used glide path files are the ProGlider (Dentsply Sirona, Ballaigues, Switzerland) with a 0.160 mm tip size and a variable taper, the Edge Glide Path (EdgeEndo, Johnson City, TN, USA) with a 0.190 mm tip size and a variable taper, and the R-Pilot (VDW, Munich, Germany) with 0.125 mm tip size and a 0.047 constant taper. Due to the multiplicity of variables that may influence the cyclic fatigue strength, an assessment of the glide path instruments’ performance using a multimethod approach [15] seems to be a reliable way of providing answers regarding the instruments’ weaknesses and strengths. Given the lack of clear information and results regarding the multiple aspects that constitute the instruments’ characteristics, such an approach may improve the methodological internal validity and provide more comprehensive knowledge.

Considering the impact that NiTi glide path mechanized instruments may have on preventing complications during root canal preparation, such as reducing the chance of instrument separation, an investigation was conducted in order to comprehensively assess the cyclic fatigue strength of three glide path files (ProGlider (Dentsply Sirona), Edge Glide Path (EdgeEndo) and R-Pilot (VDW)) through a multimethod approach [15] that addressed multiple aspects of the instruments, such as instrument design, nickel and titanium ratios and phase transformation temperatures. The null hypothesis was that there were no differences between the tested instruments’ cyclic fatigue time to fracture.

## 2. Materials and Methods

A total of 63 mechanized glide path instruments from three different systems (21 files per group) with a length of 25 mm were assessed regarding their macroscopic and microscopic design, metallurgical features, and cyclic fatigue strength. The systems included were: the ProGlider (Dentsply Sirona), Edge Glide Path (EdgeEndo), and R-Pilot (VDW). Prior to any assessment, the instruments were macroscopically checked for any visible defect that would exclude them from the test. No instrument was discarded.

### 2.1. Instrument’s Design

A stereomicroscopic analysis of the glide path instruments (*n =* 6 per group) was conducted to determine the number of blades in the active area. The average degree of the six most coronal helical angles of each instrument was also measured using the ImageJ software (Image J v1.50e, Laboratory for Optical and Computational Instrumentation, Bethesda, MD, USA) based on photos taken perpendicular to the instrument’s long axis using a camera (Canon EOS 500D, Tokyo, Japan) mounted on the operative microscope (Opmi Pico, Carl Zeiss Surgical GmbH, Oberkochen, Germany). A scanning electron microscopy (SEM) (Hitachi S-2400, Hitachi, Tokyo, Japan) analysis was conducted to investigate the blade spiral design of the instrument’s active area (symmetry and presence/absence of radial lands), to analyze the instrument’s tip (active or non-active) and cross-sectional geometry, and to identify marks linked to the machining production process, surface finishing procedures, and minor defects or deformations.

### 2.2. Metallurgical Features

A sample of three instruments per group was analyzed using energy-dispersive X-ray spectroscopy (EDS). The assessment was performed on a conventional scanning electron microscope Hitachi S-2400 (Hitachi, Tokyo, Japan) equipped with a tungsten filament electron gun and “out-lens” detectors. The instruments to be analyzed were placed on a file holder and mounted in the microscope chamber. The vacuum was created during a 10 min period. Regarding the operative setting conditions, the acceleration voltage was 20 kV, the filament current was 3.1 amperes and the work distance was 25 mm. The elemental analysis was conducted using an EDS detector (Bruker Quantax, Bruker Corporation, Billerica, MA, USA) supported by images obtained by backscattered electrons. The data acquisitions were made with a 60 s lifetime in order to optimize the image conditions. The sampling was performed on an endodontic instrument area of 400 µm × 400 µm. The final elemental analysis was semi-quantitative using ZAF correction, and the result was analyzed using the Systat software (Systat Software Inc., San Jose, CA, USA). The proportions of the titanium and nickel elements were obtained, and when available, the presence of traces of other metallic elements was documented.

Differential scanning calorimetry (DSC) tests, which followed the international guidelines’ recommendations [16], was also used to characterize the transformation temperature of the instrument’s material. Prior to the DSC tests, the samples were prepared. A 2 to 3 mm fragment was removed from each instrument’s coronal active portion, submitted to a chemical etching bath with a solution made of 25% hydrofluoric acid (HF), 45% nitric acid (HNO_3_), and 30% distilled water (H_2_O) for approximately 2 min, followed by neutralization in distilled water. Finally, the samples were weighed on an M-Power microbalance (Sartorius, Goettingen, Germany) with the optimal weight of 5 to 10 mg. Each sample was then placed in an aluminum pan of approximately 38 mg and 3 mm in diameter, while an empty aluminum pan was used as the control. A differential scanning calorimeter, DSC 204 F1 Phoenix (Netzsch-Gerätebau GmbH, Selb, Germany) was used. Each test’s thermal cycle started with 5 min at room temperature, followed by heating up to 150 °C, a 2 min stabilization plateau at the maximum temperature level, a cooling cycle until −150 °C was reached, and a 5 min stabilization plateau. Then, a new heating phase was conducted by heating up to 150 °C, followed by another 2 min stabilization plateau. Finally, the temperature decreased to room temperature, and one last 2 min stabilization plateau was completed. Both the heating and cooling phases were conducted at a rate of 10 °C per minute variation in pace. The overall DSC test lasted approximately 1 h and 40 min. The test was conducted under atmospheric gaseous nitrogen (N_2_) conditions with a flow of 20 mm per minute. The test’s final results were observed by using the Netzsch Proteus Thermal Analysis (Netzsch-Gerätebau GmbH, Selb, Germany) software, from where the phase transformation temperatures were extracted. Two tests were conducted on two instruments from the same system. The second test aimed to confirm the result of the first one.

### 2.3. Cyclic Fatigue Testing

The calculation of the sample size used the two instruments presenting the highest difference between them after six tests as a reference. Considering a power level of 80%, an alpha-type error of 0.05, and an unstandardized effect size of 213.0 ± 120.6 (ProGlider vs. R-Pilot), at least seven instruments per group would be necessary. A final sample size of ten instruments per group was chosen.

The files to be tested were placed on a 6:1 reduction handpiece powered by a Silver Reciproc motor (VDW GmbH, Munich, Germany). The handpiece was fixed on a custom-made tube model device (Odeme Dental Research, Luzerna, Santa Catarina, Brazil) that allowed us to reproduce the methodology for all instruments to be tested. The instruments were able to rotate on a stainless-steel artificial canal with a 6 mm curvature radius, 86 degrees of curvature and a 1.4 mm inner diameter, which had a length of 9 mm with the position of maximum stress load located in the middle of the curvature length. The cyclic fatigue tests were performed with a static model using glycerin as a lubricant in a continuous rotary motion at a speed of 300 rpm and torque of 3.5 N·cm (ProGlider and Edge Glide Path) or in an asymmetric oscillatory counterclockwise motion using the program RECIPROC ALL (R-Pilot). The tests were conducted at room temperature (20 °C), which is in accordance to the international guidelines for NiTi super-elastic materials tensile testing [17]. The instruments were rotated freely inside the artificial canal until fracture. The fracture moment was noted both audibly and visually, and the time to failure was recorded on a chronometer. The size of the separate fragments was determined using a digital caliper (Mitutoyo, Aurora, IL, USA).

### 2.4. Statistical Analysis

Results were expressed by both the mean and standard deviation and the median and interquartile range values. Data normality was verified using the Shapiro–Wilk test. The non-parametric Mood’s median test was selected to evaluate the helical angle and time to fracture, while the length of the separated fragments was compared using one-way ANOVA and post hoc Tukey tests (SPSS v22.0 for Windows; SPSS Inc., Chicago, IL, USA). The significance level was set at 0.05.

## 3. Results

**Figure 1:**
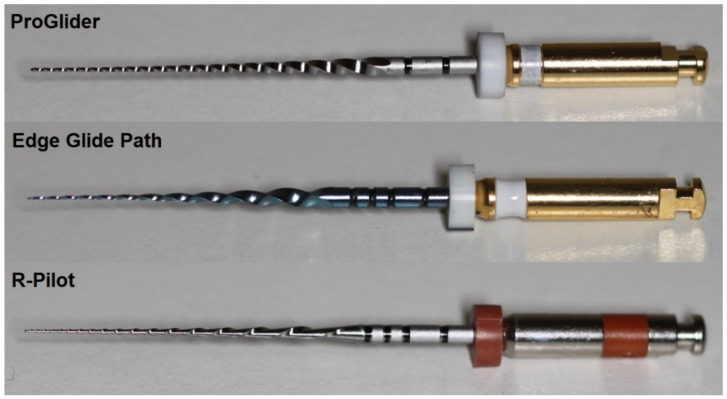
Macroscopic photos of the assessed instruments. A higher number of blades is observed in the ProGlider and R-Pilot files.

**Figure 2:**
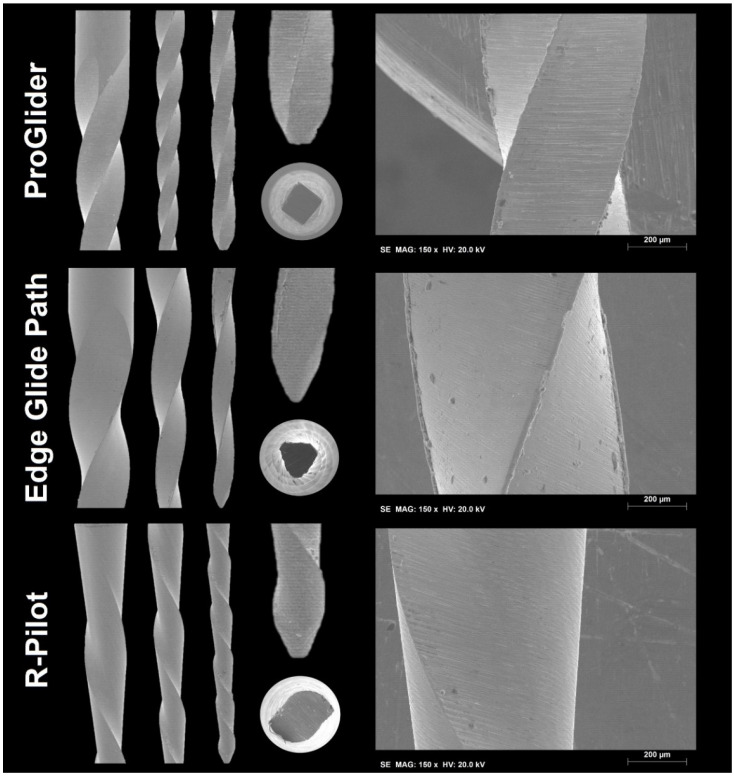
Representative scanning electron microscope images of the instruments’ blade design (left), tip and cross-section (center), and surface finishing (right). Differences are observed in the tip and cross-sectional design. The Edge Glide Path’s surface appears smoother, while the R-pilot’s surface shows less debris and metal rollovers.

The highest number of blades and the highest helical angle means were noted in the ProGlider (*n =* 21) and R-Pilot (26.4°) instruments, respectively, while the lowest were observed in the Edge Glide Path (*n =* 11, 18.4°) (Figure 1 and Table 1). The SEM analysis revealed a design of symmetrical blades without radial lands in all instruments. The tips were different between groups and non-active. Square, triangular, and S-shaped cross-section geometries were observed for the ProGlider, Edge Glide Path, and R-Pilot instruments, respectively. At high magnifications, parallel marks originating from the instruments’ manufacturing process were observed in the tested files, although the Edge Glide Path presented the smoothest surface. The R-Pilot system had the least visible debris and metal rollover defects (Figure 2).

**Figure 3:**
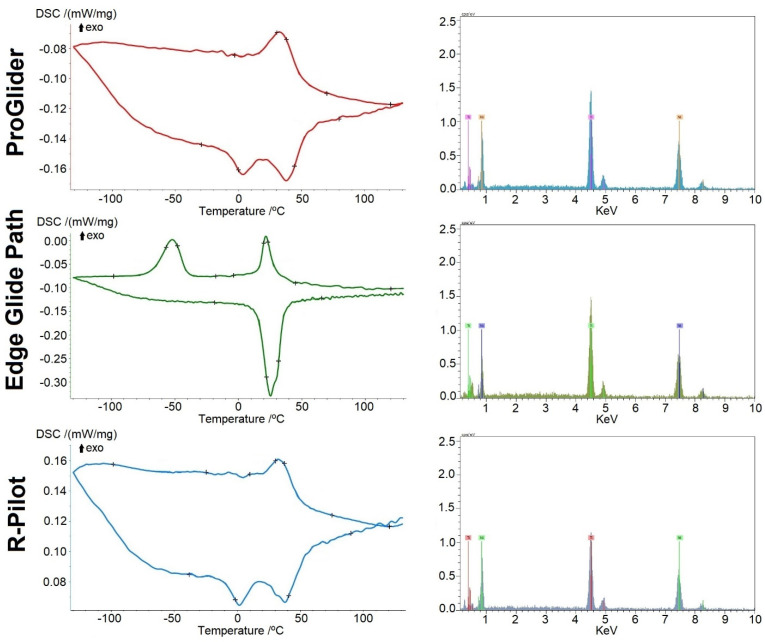
Differential scanning calorimetry (left) and energy-dispersive X-ray spectroscopy (right) charts. The differential scanning calorimetry charts from the ProGlider and R-Pilot instruments have some similarities in both the cooling (top) and heating (bottom) curves, while the Edge Glide Path is distinct. The energy-dispersive X-ray spectrometers are equivalent among all instruments.

All instruments are made from NiTi alloys with an almost equiatomic Ni/Ti ratio (Figure 3 and Table 1). The highest and lowest R-phase start (Rs) were observed in R-Pilot (50.3 °C) and Edge Glide Path (28.3 °C), respectively, while the highest and lowest R-finish (Rf) were noted in R-Pilot (21.2 °C) and ProGlider (13.7 °C), respectively (Figure 3 and Table 1). In the cyclic fatigue testing, the highest fracture time was observed in R-Pilot (356.6 s), while the lowest was detected in Edge Glide Path (111.0 s) (*p* < 0.05). All fragment lengths were equivalent (*p* > 0.05) (Table 1).

## 4. Discussion

Over the past few years, several studies have analyzed the cyclic fatigue strength of multiple instruments with the sole purpose of reporting the data [18,19]. Although this is a valid approach, it lacks a more comprehensive methodology beyond laboratory testing, which does not mimic clinical activity and only provides numeric parameters for mechanical strength that may serve as a reference to assume a greater or lesser capacity to withstand cyclic stress before instrument separation. Even though this reference has been considered valuable over the years, a more comprehensive multimethod approach has been advocated to counterbalance the simplicity of such tests [15] by complementing the mechanical tests with the assessment of multiple qualitative data. The present study included the inspection of the design and metallurgical characteristics, which provided data on the instruments’ behavior during the cyclic fatigue test and reported significant differences among instruments, therefore rejecting the null hypothesis.

Due to the great diversity in design and geometry, it is difficult to interpret the mechanical results without considering each instrument’s characteristics. The superior number of blades per millimeter gives the ProGlider and R-Pilot higher flexibility, leading to a superior cyclic fatigue strength when compared to the Edge Glide Path [20,21]. Moreover, although the Edge Glide Path’s smoother surface finishing tends to give it an advantage over the others with more irregular surfaces, the higher presence of debris and metal rollover defects may work oppositely [22]. The R-Pilot showed the least debris and metal rollovers, which tends to improve this instrument results. The cross-section geometries were all different but followed the manufacturers’ description with the Edge Glide Path’s and R-Pilot’s triangular and S-shaped geometries giving them smaller cross-sectional core diameters, and contributing to their superior cyclic fatigue strength over the ProGlider’s square design [20]. Additionally, the martensitic characteristics of R-Pilot at test temperatures tend to increase its time to fracture compared to the mixed austenite plus R-phase of both the ProGlider and Edge Glide Path [23,24]. Although these combined characteristics could have an influence on the final results, it is not possible to determine how much each characteristic influences the results, or if it has more or less influence than the others.

All these characteristics combined and associated with the kinematics [10], which is probably one of the most relevant characteristics, lead the reciprocating R-Pilot to have a significantly higher time to fracture than both the other rotation systems. These results partially corroborate previous studies where both the R-Pilot and Edge Glide Path were superior to ProGlider [25,26]. Although direct comparisons are hindered by the different test settings [27], these non-corroborating previous results for the Edge Glide Path vs. ProGlider should not be seen as inconsistent due to the high internal validity of cyclic fatigue testing. Lee et al., who noted differences between the ProGlider and Edge Glide Path [25] reported a mean fragment length between 2.33 mm (Edge Glide Path) and 2.82 mm (ProGlider), while the lengths in the present study were 7.0 mm and 7.1 mm due to the tests being conducted at different file positions. The fact that Lee et al. [25] noticed a significant difference between files does not contradict the present study since it can be partially explained by the multi-taper characteristics of both instruments, which may not necessarily match, and despite the setting differences, they could end up complementing each other. Additionally, and although not assessed in the present study, previously published SEM analysis of the fractures’ surfaces of these same three tested instruments detected dimples and fatigue striations that characterize cyclic fatigue failure [25,28].

The present research design follows a previously published methodology [29] that reported on the same three groups of glide path instruments. The study assessed the maximum load under a bending test and noted that the ProGlider instruments showed the lowest outcomes (145.7 gf) (higher flexibility), followed by R-Pilot (164.8 gf), while Edge Glide Path presented the highest maximum load (329.9 gf) (lower flexibility). Those results are in agreement with the present study since higher flexibility is usually associated with higher time to fracture in cyclic fatigue testing, which was observed, in terms of total results, for ProGlider (119.5 s) compared to Edge Glide Path (111.0 s).

Several studies have advocated the use of body temperature in cyclic fatigue testing with the argument that it better mimics clinical conditions [30,31]. Although the simplicity of the cyclic fatigue test hardly represents any relevant clinical action regardless of the settings that may be used, it is also true that test temperature may indeed have an influence on the behavior of some types of instruments [32,33]. Previous studies have advocated the use of phase transformation temperature analysis [15,34] in order to more comprehensively address the influence of temperature on the outcomes by assessing a service temperature window that may range from storing room temperature to body temperature, as opposed to the rigidity of a fixed body temperature testing.

The present study ran the mechanical tests at room temperature in order to set the baseline outcomes, which is in accordance with the norms for conducting tension tests on NiTi super-elastic materials, which suggest testing at 22 °C ± 2 °C [17] unless otherwise specified. These tests were comprehensively complemented with the phase transformation temperature analysis, which was also conducted in accordance to international standards [16]. Considering the heating curves in the DSC chart (Figure 3), both the ProGlider and R-Pilot instruments are in an austenitic plus R-phase crystallographic arrangement at both room and body temperature. On the other hand, the Edge Glide Path files are in an austenitic plus R-phase state at room temperature but they are almost fully austenitic at body temperature. This may suggest a higher stiffness (lower flexibility) of the latter instrument, when compared to the others. Due to the influence of the body temperature, the temperature of the instrument tends to rise during the root canal preparation, although several other factors may affect this temperature rise [32], such as the irrigants’ temperature. Such a change in temperature may affect the in-service real crystallographic arrangement of the Edge Glide Path files, which may be clinically relevant depending on the amount of re-arrangement (partial or full) that may happen in a real clinical practice setting. This is not easily determinable at the laboratory level.

The main strengths of the present study are that it complies with international standard guidelines [16,17] and/or well-accepted previously published methodologies [15,34]. A 6 mm radius and 86 degrees of curvature was used in order to allow for a severe curvature capable of inducing a superior stress [15,34]. Additionally, the multimethod research applied to the cyclic fatigue testing allowed for a broader understanding of the results [15], by assessing parameters that have been previously considered relevant to the instrument’s mechanical performance such as the file design (assessed using stereomicroscopy and SEM) [21], the constitution of the elements (EDS) [23], or the crystallographic arrangement at test temperature (DSC) [4,23,24]. However, the laboratory cyclic fatigue results are still of limited use when aiming to understand the instrument’s true in vivo clinical strength when preparing for a real root canal. Future studies should aim to determine the fracture incidence of these instruments in clinical practice, or by assessing, in ex vivo conditions, the clinical efficacy of these instruments when performing canal shaping procedures through micro-CT assessment.

## 5. Conclusions

The present multimethod analysis showed that the assessed instruments differ in the number of blades, helical angle means, the instrument’s cross-section, surface finishing, and phase transformation temperatures. Although the multimethod assessment allowed us to comprehend that each instrument is unique with some characteristics that favor, and others that harm the cyclic fatigue results, in general it can be concluded that the reciprocating R-Pilot instrument has the capacity to sustain a significantly higher amount of cyclic fatigue stress when compared to the ProGlider and Edge Glide Path rotary files.

## Figures and Tables

**Table 1:** Instrument’s design, metallurgical features and cyclic fatigue results (mean (standard deviation) and median (interquartile range)).

System	Instrument Design		Cyclic Fatigue		Metallurgic Features				
	Number of Blades	Helical Angle Mean [Range]	Time to Fracture (s)	Fragment Length (mm)	Cooling		Heating		Elements Composition (Ni/Ti Ratio)
					Rs (°C)	Rf (°C)	As (°C)	Af (°C)	
ProGlider	21	21.1° ^a^ (20.1°–21.7°)	119.5 (±12.9) ^a^ 123.5 (109.5–129.8)	7.0 (±0.2) ^a^ 7.1 (6.9–7.1)	50.1	13.7	−9.6	54.9	1.035
Edge Glide Path	11	18.4° ^a^ (16.6°–21.3°)	111.0 (±42.9) ^a^ 113.5 (73.3–152.8)	7.1 (±0.6) ^a^ 7.1 (6.7–7.5)	28.3	16.0	15.1	36.2	1.003
R-Pilot	17	26.4° ^b^ (24.8°–30.6°)	356.6 (±40.9) ^b^ 353.5 (321.8–378.5)	6.8 (±0.6) ^a^ 7.0 (6.7–7.2)	50.3	21.2	−11.0	54.8	1.031

Different superscript letters represent statistically significant differences (*p* < 0.05) between instruments. Rs = R-phase start; Rf = R-phase finish; As = Austenitic start; Af = Austenitic finish.

## Data Availability

Not applicable.
